# Comment on: Corneal collagen cross-linking with riboflavin and ultraviolet: A light for keratoconus

**DOI:** 10.4103/0301-4738.64118

**Published:** 2010

**Authors:** Anand Parthasarathy

**Affiliations:** Vasan Eye Care Hospital, 120 A Bazaar Road, Saidapet, Chennai-600 041, Tamil Nadu, India

Dear Editor,

I read with interest the article by Agrawal on the results of "Corneal collagen cross-linking with riboflavin and ultraviolet – a light for keratoconus: results in Indian eyes".[1] The author has done commendable work and the results do definitely add to the present body of evidence. However, I seek clarifications from the author.

Was the presence of vernal keratoconjunctivitis (VKC) specifically looked for as we know that there is an association between VKC and keratoconus?[2‐4]

The author mentions in the results section that the best corrected visual acuity (BCVA) improved or remained stable in 54% (20/37) and 28% (10/37) respectively of the eyes. Would the authors state what the result in the remaining seven eyes was? Did the patients have loss of BCVA, and if so, why? Did these patients have VKC?

In addition to the Orbscan, postoperative ultrasound pachymetry and topography should also have been performed to assess the change in the corneal thickness. This would be better than an Orbscan in assessing the change of K values. This is important since any scanning slit-based imaging technology would be affected by the stromal haze that appears after collagen cross-linking in these patients.

In the results section of the article, Table 1 shows values and change of Kmax Apex, Kmax (D), astigmatism (D) and visual acuity with large standard deviations. This could indicate variability in the response of the procedure, increase in the keratometric values in astigmatism and drop in visual acuity in some patients. The K-values decreased in 66% (24/37) of the eyes and were stable in 22% (8/37) of the eyes; five of the patients had an increase in keratometry. The author would do well to allude to it further.

The details of the wavefront analysis should have been elaborated further unless it is a subject of another study. Studies performed at our centers indicated that wavefront analysis can be used as an additional tool to detect forme fruste in keratoconus patients if the wavefront decomposition is performed up to the 4th order. I would encourage the author to share information if he has details regarding the same.
